# The human solute carrier family 11 member 1 protein (SLC11A1): linking infections, autoimmunity and cancer?

**DOI:** 10.1111/j.1574-695X.2007.00231.x

**Published:** 2007-03

**Authors:** Agnes A Awomoyi

**Affiliations:** Department of Microbiology and Immunology, University of Maryland School of Medicine, University of Maryland Baltimore, MD, USA

**Keywords:** SLC11A1, cancer, infections, autoimmunity, chronic inflammation, ATF/CREB

## Abstract

SLC11A1 is known to link infections, autoimmunity and cancers. A review is presented of the mechanisms by which a balance is maintained between infections caused by pathogens (viral, bacterial and protozoan; intracellular and extracellular) and disorders resulting from (acute or chronic) inflammation, and of the interactions that determine how the initial innate immune system directs subsequent acquired immune responses in human populations

This review discusses the role of the solute carrier family 11 member 1 protein (SLC11A1), formerly NRAMP1 (for natural resistance associated macrophage protein 1), in linking infections, autoimmunity and cancer. Chronic inflammation is caused by a variety of factors including bacterial, viral and parasitic infections, chemical irritants and nondigestible particles. Chronic inflammation is associated with a predisposition to cancer and autoimmunity because the longer the inflammation persists, the higher the risk of associated autoimmunity and carcinogenesis. Immune cells involved in inflammation include leucocytes such as neutrophils, monocytes, macrophages and eosinophils. These cells provide the soluble factors that are thought to mediate the development of inflammation. When inflammation goes unchecked it often leads to chronic inflammation-associated diseases. *Slc11a1* in mice encodes a polytopic integral 10–12 transmembrane protein, which is expressed exclusively in macrophages and polymorphonuclear leukocytes and neurons (Evans *et al.*, 2001). Slc11a1 exacts pleiotropic effects on macrophage function that include enhanced chemokine KC, tumor necrosis factor-α, interleukin-1β, inducible nitric oxide synthase and MHC class II expression; all are important in the induction and maintenance of autoimmunity and cancer but also are crucially important in resistance to intramacrophage pathogens such as tuberculosis.

The protein is localized to the acidic endosomal and lysosomal compartment of the macrophage (Searle *et al.*, 1998). In resting macrophages, slc11a1 resides within the phagolysosome, but upon activation of the macrophage slc11a1 translocates to the membrane of the phagolysosme and serves as a divalent metal cation transporter regulating and regulated by cellular iron (fe ^2+^) levels (Atkinson & Barton, 1988; Atkinson *et al.*, 1997). Under normal physiological conditions, slc11a1 delivers bivalent metal cations from the cytosol into acidic late endosomal and lysosomal compartment where the Fenton/and or Haber–Weiss reactions generate toxic antimicrobial radicals for direct antimicrobial activity against phagocytosed microorganisms (Goswami *et al.*, 2001). Prolonged accumulation of toxic radicals can, however, have detrimental effects, causing cell or tissue damage and contributing to the development or progression of numerous diseases including cancer and autoimmunity. Toxic radicals generated as a result of macrophage activation in response to stimuli are reactive oxygen (generically referred to as oxidants: superoxide, hydrogen peroxide, hypochlorous acid, singlet oxygen, and the hydroxyl radical) and nitrogen (nitric oxide by the inducible nitric oxide synthase) intermediates (Schumacker, 2006). Exogenous and endogenous agents that mediate inflammation by activating the macrophage can cause slc11a1 to translocate to the membrane of the phagolysosome, where it serves as a cation transporter. Activated macrophage-derived hydrogen peroxide may come into contact with transition metals transported by slc11a1 into lysosome or on DNA/RNA in the late endosomal compartments or may diffuse into the nucleus, forming a radical and giving rise to the strand breaks and base modifications that eventually lead to preneoplastic alterations (Storz, 2005). Thus, slc11a1 functions to transport metal ions from high (cytoplasmic) to low (intravascular) pH.

It is possible that the significant increase in iron deposition observed in the synovial membrane of rheumatoid arthritis patients, and foam cells in atherosclerotic lesions, may be attributable to SLC11A1. SLC11A1 might contribute to the pathogenesis of human systemic lupus erythematosus, because particulate auto-antigens containing nucleic acid and proteins released from dying or damaged cells can be recognized by B cells that have been TLR 3, 7, 8, and 9-activated and differentiated into antibody-producing plasma cells (Leadbetter *et al.*, 2002; Rifkin *et al.*, 2005). In mice, slc11a1-positive phagosomes during *Mycobacterim bovis* infection are significantly more acidic than those formed in slc11a1-negative ones (Hackam *et al.*, 1998).

The *SLC11A1* gene, located on human chromosome 2q35, is *c*. 14 kb in length and contains 15 exons. In humans, a microsatellite polymorphism with a Z-DNA-forming dinucleotide repeat in the 5′ terminal region of the *SLC11A1* is associated with infections, (ranging from viral to protozoan; Shaw *et al.*, 1997; Bellamy *et al.*, 1988; Marquet *et al.*, 1999; Cervino *et al.*, 2000; Gao *et al.*, 2000; Meisner *et al.*, 2001; Awomoyi *et al.*, 2002; Ryu *et al.*, 2002; Liu *et al.*, 2004; Mohammed *et al.*, 2004; Shaw *et al.*, 1997), autoimmunity (such as Kawasaki disease, sacoidosis, juvenile rheumatoid arthritis, rheumatoid arthritis, inflammatory bowel disease (IBD)), and diabetes; most of these diseases are thought to have a microbial origin; (Hofmeister *et al.*, 1997; Maliarik *et al.*, 2000; Sanjeevi *et al.*, 2000; Singal *et al.*, 2000; Yang *et al.*, 2000; Kojima *et al.*, 2001; Ouchi *et al.*, 2003) multiple sclerosis (Kotze *et al.*, 2001) and more recently with esophageal cancer in a South African coloured population (Zaahl *et al.*, 2005).

Examination of the pattern of genetic diversity at eight microsatellite markers within a 3-Mb region flanking the SLC11A1 locus across geographically diverse human populations has provided evidence for genetic differences at the SLC11A1 locus in 21 global human populations (Awomoyi & Tishkoff, unpublished data).

Because SLC11A1, protein translocates to the membrane of the late endocytic/lysosomal compartment upon activation of the macrophage (Gruenheid *et al.*, 1997), serves as an antiporter fluxing cations in either direction of the membrane (Goswami *et al.*, 2001), namely into the phagolysosome or into the cytosol depending on the pH gradient, it should influence the assembly of the inflammasome complex and affect the ability of the macrophage to secrete pro-inflammatory interleukin (IL)-1β and IL-18. It should influence antiviral responses mediated by the cytosolic helicases RIG-I (for retinoic acid-inducible protein I) and MDA5 (for melanoma differentiation-associated protein 5). Apart from being involved in inflammatory activities, SLC11A1 might influence apoptotic processes by regulating the function of cytosolic nucleotide – oligomerization domains (NODs), a group of proteins of which cryopyrin, a constituent of the inflamasome complex, is a member. Loss or gain of function of the NOD proteins is associated with several disregulated immune response disorders (Masumoto & Inohara, 2005). Transcriptional activation of *SLC11A1* should lead to apoptosis, in accordance with the notion that genes that deplete the iron content of the cell cytosol antagonize cell growth. By contrast, transcriptional repression of *SLC11A1* could lead to cell survival. SLC11A1 might influence immune responses to viral vector delivery systems (Thorsen *et al.*, 2006; Xu *et al.*, 2006; Zhang & Godbey, 2006), for example herpes virus vector delivery system for CpG, adenovirus vectors double-stranded RNA, retroviral vectors; single-stranded RNA, adenovirus associated viral vectors single-stranded DNA and baculoviral vectors, by regulating antigen processing and the activity of proteases in the late endosomal compartment.

Finally, intracellular bacteria such as attenuated strains of *Salmonella typhimurium* are widely used as vehicles for delivery and expression of vaccine antigens in infectious (Soo *et al.*, 1998) and noninfectious disease models (Barnett *et al.*, 2005; Kalvakolanu DV, pers. commun., unpublished information). In mice, early control of bacterial replication following infection with *S. typhimurium* is controlled by slc11a1. SLC11A1 might influence the responses to such recombinant bacteria vaccines in multiple ways, by, for example, regulation of bacterial load or recombinant antigen dose, class II molecule expression, costimulatory or adjuvant activity, or antigen processing by regulation of the activity of proteases in the late endosomal compartment. Results in congenic mice using live attenuated *S. typhimurium* aroA aroD mutants carrying recombinants antigen tetanus toxoid, and leishmanial gp63 showed that wild-type *S. typhimurium*-resistant mice mounted a predominantly Th1-type response to vaccination, whereas mutant mice mounted a predominantly Th2-type response (Lang *et al.*, 1997; Soo *et al.*, 1998). Analogous to this in humans are the two predominant SLC11A1 (GT)_n_ Z-DNA promoter alleles, namely allele 2 and allele 3. These alleles are reported to account for opposing levels of SLC11A1 gene expression: the allele t(gt)_5_ ac (gt)_5_ ac (gt)_9_ g (also known as allele 3) drives high gene expression, while the allele t(gt)_5_ ac (gt)_5_ ac (gt)_10_ g (also known as allele 2) drives low gene expression (Searle & Blackwell, 1999). In addition, the allele that drives high gene expression is associated with autoimmunity and cancer but protects against infectious diseases, whereas the one that drives low expression is associated with infections such as tuberculosis but protects against autoimmunity and cancer. These observations suggest that chronic hyperactivation of macrophages associated with allele 3 is functionally linked to cancer and autoimmune disease susceptibility, while the poor level of SLC11A1 expression promoted by allele 2 contributes to infectious disease susceptibility.

A summary of the distribution of these alleles based on genotyping shows that, whereas the level of distribution of allele 2 is *c*. 0.176% in the Nigerian population and 0.104% in the Gambian population, it is a little higher in East African and non-African populations: *c*. 0.268% in the Tanzanian population, *c*. 0.24% in the Lebanese population, 0.3% in European populations, and 0.265% in New World populations. The level in the Chinese population (Awomoyi & Tishkoff, unpublished data), at 0.1%, is similar to that in the African populations (Table 1). Allele 2 is the intracellular infection susceptibility allele. It could be the case that in endemic infectious disease populations the protective allele 3 is maintained at a higher frequency. Given the important role of this gene as a cation antiporter influencing macrophage function, it can be speculated that, in humans, populations with genetic differences in haplotype structure and linkage disequilibrium (LD) patterns will exhibit altered susceptibility to disease and will respond differently to infections and therapy, whether curative or prophylactic. This could impact greatly on vaccine delivery and efficacy (Awomoyi & Tishkoff, unpublished data).

**Table 1:** Distribution of SLC11A1 Z-DNA promoter alleles in global human populations

Population	Chromosome numbers (2n)	Allele 1	Allele 2	Allele 3	Geographic/country region	Geographic region allele frequency
Wollof	24	2 (0.085)	2 (0.085)	20(0.83)	The Gambia	
Mandinka	54	1 (0.02)	2 (0.04)	51 (0.94)		Allele 1: 0.053, 2: 0.104, 3: 0.843.
Gambian/other	48	2 (0.05)	5 (0.01)	41 (0.85)		
Fula	34	2 (0.06)	5 (0.015)	27 (0.79)		
Igala	46		5 (0.11)	41 (0.89)	Nigeria	
Bassange	28		7 (0.25)	21 (0.75)		
Ibo	52		6 (0.12)	46 (0.88)		Allele 1: 0.005, 2: 0.176, 3: 0.819.
Fulani	40		9 (0.225)	31 (0.775)		
Koma	94		17 (0.18)	77 (0.82)		
Yoruba	106	3 (0.03)	18 (0.17)	85 (0.801)		
Malawians1	2542	48 (0.019)	524 (0.206)	1970 (0.775)	Malawi	Allele 1: 0.019, 2: 0.206, 3: 0.775
Burunge	38		9 (0.24)	29 (0.76)	Tanzania	
Hadza	42		7 (0.17)	35 (0.83)		
Maasai	46		16 (0.35)	30 (0.65)		Allele 2: 0.268, 3: 0.732.
Sandawe	50		17 (0.34)	33 (0.66)		
Turu	38		9 (0.24)	29 (0.76)		
South Africans	1284	11 (0.008)	361 (0.281)	912 (0.71)	South African Caucasians	Allele 5: 0.008, 2: 0.281, 3: 0.71
Lebanesse	68	1 (0.02)	16 (0.24)	51 (0.75)	Europe/Middle East/Asia	
N. Europe	20		8 (0.4)	12 (0.6)		Allele 1: 0.002, 2: 0.235, 3: 0.763
Russian	20		4 (0.2)	16 (0.8)		
Chinese	20		2 (0.1)	18 (0.9)		
San Martin	50		12 (0.24)	38 (0.76)	New World	Allele 1: 0.01, 2: 0.265, 3: 0.725
Quechua	48	1(0.02)	14 (0.29)	33 (0.69)		

Recent reports show that it is the type and concentrations of immuno-modulators present at the site of infection that determine the outcome of infection. For example, upon macrophage activation the presence of IL-6 alone (Rochman *et al.*, 2005), IL-6 together with TGF-beta (Veldhoen *et al.*, 2006a, b), or TGF-beta alone (Marie *et al.*, 2002) should determine which type of T-helper acquired immune cells are activated. Induction of IL-6 alone is shown to promote the production of prostaglandins, which are important in the generation of oxidative radicals (Rochman *et al.*, 2005). IL-6 produced in combination with TGF-beta is important in driving the differentiation of naive T cells to the pro-inflammatory T-helper 17 phenotype that produces IL-17 (Veldhoen *et al.*, 2006a, b). IL-17 is associated with chronic inflammation and therefore autoimmunity in several experimental animal models. TGF-beta alone may induce anti-inflammatory forkhead box protein (foxp)-3 expressing regulatory T cells (FOXP) 3 ^+^T_regs_ in the periphery (Marie *et al.*, 2002).

Another class of T regulatory cells that lack the Fox3p develops under the control of persistently high levels of IL-10 (Groux *et al.*, 1997). This class of low proliferative T regulatory cells suppress antigen–specific responses and actively down regulate a pathological immune response *in vivo*. In our previous study we showed that allele 2 of the SLC11A1 promoter gene polymorphism influences IL-10 production and individuals with active TB produce higher levels IL-10 even after recovery from TB in relations to ethnically matched age and gender controls (Awomoyi *et al.*, 2002).

Agents that activate macrophage together with host SLC11A1 alleles will exert different effects on SLC11A1 function, but how SLC11A1 functions help determine the outcome of infection by modulating the production of these cytokines in not known. Comparative analysis of the *SLC11A1* genomic region in nonhuman and human primates reveals a protein with a highly conserved amino-acid sequence across species but with striking nucleotide differences at the 5′ and 3′ untranslated regions (UTRs) and within intronic regions, thus suggesting that this gene might be differentially regulated in different species. One major difference at this locus between species includes the type and locations of transcription factor binding sites in the 5′UTR of *SLC11A1*. Of particular importance is the binding site for the activating transcription factor-3 (ATF-3; also known as an AP-1-like element, Lee *et al.*, 2000) adjacent to the 5′ sequence of the Z-DNA-forming microsatellite polymorphism in humans, which is not found in mice. Figure 1 shows an alignment of portions of the SLC11A1 5′ promoter genomic region in five mammals, demonstrating that, whereas a TGACTCT ATF-3 putative binding site is present in *Homo sapiens*, *Pan troglodytes* and *Macaca mulatta*, this sequence is absent in *Mus musculus*. In *Rattus norvegicus* and *Pongo pygmaeus*, the second cytosine is replaced by a thymine. ATF-3 is a negative regulator of TLR4 (Gilchrist *et al.*, 2006). ATF-3 can heterodimerize with Jun proteins to activate transcription. By contrast, the homodimer ATF-3 on its own is a repressor of transcription when bound to DNA. ATF-3 is a member of the ATF/CREB family of basic leucine zipper (b-zip)-type transcription factors, and its expression is increased by various pathophysiological conditions and in several cancer cells (Hai *et al.*, 1999). The expression of ATF-3 is relatively low in most cell types under normal conditions but is strongly induced in response to many environmental changes as an immediate early-response gene. This B-DNA-forming sequence motif is found in the promoter of rat tumor suppressor gene (p53) (Lee *et al.*, 2000; Kawauchi *et al.*, 2002; Yau *et al.*, 2002) and human transforming growth factor beta (TGF-β) gene (Owen & Ostrowski, 1990), where it has been demonstrated to be biologically active. Similarly, it is adjacent to the 5′ promoter Z-DNA-forming microsatellite repeat polymorphism in *SLC11A1* but it is unclear whether it is an active ATF-3 binding site in SLC11A1. The formation of Z-DNA structure is known to play an active role in modulating inhibitory chromatin structure (Ha *et al.*, 2005; Liu *et al.*, 2006). Should the ATF-3 binding site be a transcriptional active site in the promoter of SLC11A1, carriage of different alleles interrupted by mutations at the adjacent Z-DNA microsatellite repeat may influence chromatin remodelling and accessibility of transcription factors. We can speculate that, under normal physiological conditions, when the homodimer ATF-3 binds to this target sequence in the 5′ promoter region of *SLC11A1* it should repress transcriptional activation of *SLC11A1*; conversely, heterodimerization of ATF3 with other transacting factors should promote transcriptional activation of SLC11A1.

**Fig. 1:**
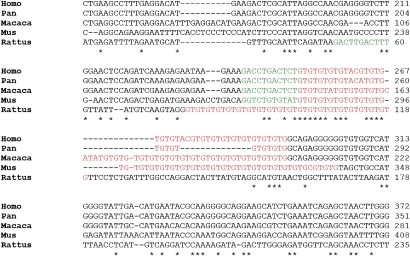
Alignment of the 5′ promoter region of SLC11A1 in five mammalian species. Alignment of *Homo sapiens*; GenBank accession number AF229163, *Pan troglodytes*; ensembl, *Macaca mulatta*; XM_001089328, mouse; GenBank accession number NM_013612, & *Rattus norvegicus*; ensembl).

It could be that transcriptional activation of SLC11A1 leads to apoptosis and cell death while transcriptional repression of SLC11A1 leads to cell proliferation and survival if unchecked could result in cancer and autoimmunity. The ATF-3 putative site in the promoter of SLC11A1 along with other transacting factors might have a regulatory role in modulating the effects of SLC11A1. This review has thus highlighted some of the mechanisms by which SLC11A1 might be mediating, influencing or modulating the outcome of these diseases and disorders in human populations.

## Statement

Re-use of this article is permitted in accordance with the Creative Commons Deed, Attribution 2.5, which does not permit commercial exploitation.
